# Incidental Subependymoma of the Floor of the Fourth Ventricle: A Case Report

**DOI:** 10.7759/cureus.103355

**Published:** 2026-02-10

**Authors:** Brooklyn K Brekke-Kumley, Pamela R Kinder

**Affiliations:** 1 Department of Clinical Education, Rocky Vista University College of Osteopathic Medicine, Billings, USA

**Keywords:** benign brain tumor, conservative managment, incidental radiological finding, migratory parasthesia, subependymoma

## Abstract

Subependymomas are rare, slow-growing intraventricular tumors that are typically discovered incidentally and seldom present with transient sensory symptoms in young adults. We report the case of a 22-year-old woman who presented with episodic migratory paresthesias and was found on brain MRI to have a small fourth-ventricular lesion radiographically consistent with subependymoma. The patient’s sensory symptoms resolved spontaneously, and no further episodes occurred. Conservative management with serial imaging was pursued. At the six-month follow-up, the lesion remained stable, and the patient was asymptomatic. Subependymomas most commonly become symptomatic due to cerebrospinal fluid obstruction, whereas episodic migratory paresthesias are unusual. This case highlights the value of early MRI in young adults with atypical sensory symptoms, which may reveal incidental yet clinically significant findings and help avoid unnecessary invasive testing. Small, non-obstructive fourth-ventricle subependymomas can be safely managed with observation, demonstrating excellent short-term clinical and radiographic outcomes.

## Introduction

Subependymomas are rare, slow-growing WHO grade I ependymal tumors that account for approximately 0.2-0.7% of intracranial neoplasms and most commonly arise in the fourth and lateral ventricles [1,2]. They typically present in the fourth or fifth decade of life, with a slight male predominance, and are frequently detected incidentally as small, well-circumscribed nodules on MRI [1,3]. Their extremely slow growth and limited parenchymal invasion contribute to their frequent incidental detection. In particular, lesions arising in the fourth ventricle tend to produce symptoms only when cerebrospinal fluid pathways are compromised, which helps explain their often indolent presentation. On imaging, subependymomas are typically well-circumscribed intraventricular lesions with benign radiographic features and a low propensity for obstruction [4,5]. When symptomatic, clinical manifestations generally relate to mass effect or cerebrospinal fluid (CSF) outflow obstruction, including headache, gait disturbance, or cranial neuropathies [2,4]. Transient or migratory sensory symptoms, such as hemifacial or limb paresthesias, are unusual and rarely associated with these tumors [6-8].

Given their indolent nature and excellent prognosis after gross-total resection, observation is increasingly recognized as an appropriate initial strategy for small, non-obstructive lesions in asymptomatic patients [6,9]. In such cases, the potential risks of surgery, including neurological morbidity related to ventricular and brainstem-adjacent anatomy, must be carefully weighed. In asymptomatic, non-obstructive lesions, these risks may outweigh the benefits of resection. In young adults, episodic paresthesias more often prompt evaluation for demyelinating, vascular, or metabolic disease rather than intraventricular neoplasms [10,11].

## Case presentation

A 22-year-old woman with no significant past medical history presented with three discrete episodes of transient sensory disturbances over four months. The first episode involved sudden-onset bilateral numbness affecting the flexor and extensor surfaces of both hands, sparing the wrists. The paresthesias persisted for approximately 16 hours before partially resolving, leaving mild residual tingling. She reported no associated weakness, visual changes, or dysarthria.

Three months later, she experienced two separate events. The first began with numbness in the left V2 facial distribution, spreading to the lower jaw, lips, tongue, and left upper limb. Sensory symptoms crossed to the right upper limb and were accompanied by approximately 30 seconds of right facial weakness. Later that afternoon, she had a second episode characterized by numbness of the right forearm descending to the hand, initially affecting the little finger and migrating to the thumb. This episode was followed by recurrent paresthesias in the V2-V3 facial distribution, lips, and tongue, along with another brief right facial paresis. Both episodes lasted roughly one hour and were followed by a mild, short-lived headache.

**Figure 1:**
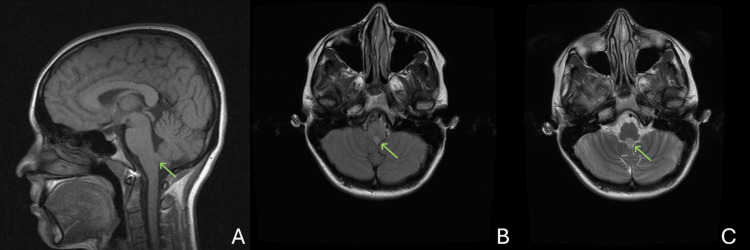
Initial MRI demonstrating a small fourth-ventricular lesion A. Sagittal T1-weighted image showing a 7 x 7 x 11 mm, well-circumscribed nodule along the floor of the fourth ventricle (arrow), without mass effect. B. Axial T1-weighted image demonstrating iso- to mildly hypointense signal of the lesion relative to gray matter (arrow). C. Axial T2-weighted postcontrast image showing mild hyperintensity and subtle but present mild enhancement of the lesion (arrow), without surrounding edema or hydrocephalus.

Between attacks, neurological examination was normal, including cranial nerves, strength, sensation, coordination, reflexes, and gait. Laboratory evaluation, including complete blood count (CBC), comprehensive metabolic panel (CMP), thyroid-stimulating hormone (TSH), lipid panel, hemoglobin A1C, and vitamin B12, was unremarkable. Brain MRI sequences included T1-weighted pre- and post-contrast, T2-weighted, and fluid-attenuated inversion recovery (FLAIR) imaging, which revealed a well-circumscribed 7 × 7 × 11 mm nodule embedded in the floor of the fourth ventricle (Figure 1A-1C). The lesion was T2-hyperintense with subtle but present mild, heterogeneous enhancement and no associated hydrocephalus or mass effect. Diffusion-weighted imaging was also performed and was unremarkable. EEG was not performed, given the lack of seizure-like features, and vascular imaging (magnetic resonance angiography/magnetic resonance venography) was not obtained due to low clinical suspicion for transient ischemic events. Given the lack of mass effect or CSF obstruction, the lesion was considered an incidental finding rather than the direct cause of the patient’s transient symptoms. Radiographically, the findings were consistent with subependymoma.

**Figure 2:**
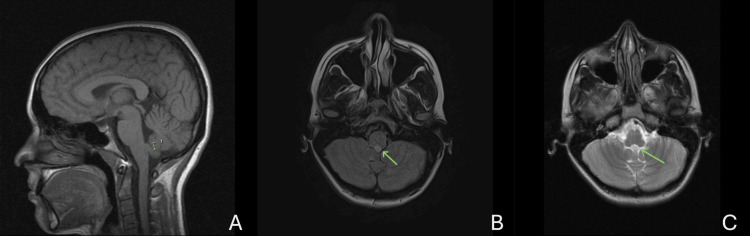
Six-month follow-up MRI demonstrating radiographic stability A. Sagittal T1-weighted image showing unchanged size and location of the fourth-ventricular lesion (bracket). B. Axial postcontrast T1-weighted sequence showing no interval enhancement or mass effect from the lesion (arrow). C. Axial T2-weighted postcontrast image demonstrating stable signal characteristics without new obstruction or edema (arrow).

Given the lesion’s small size, benign imaging characteristics, and the patient’s asymptomatic status between events, a multidisciplinary team recommended conservative management with close clinical and radiographic follow-up. The patient was counseled regarding the natural history of subependymomas, potential warning signs, and the low risk of acute complications. At six-month follow-up, the lesion remained stable on MRI, and the patient reported no recurrence of sensory episodes (Figure 2A-2C). While MRI at six months demonstrated stability of the lesion, longer-term follow-up is required to confirm enduring indolence.

## Discussion

Subependymomas are typically incidental, slow-growing intraventricular tumors, with symptoms most often arising from CSF obstruction rather than focal neurological deficits [1,2,4]. In contrast, the present case involved episodic, migratory sensory symptoms without radiographic evidence of hydrocephalus or mass effect, highlighting an atypical clinical context in which such lesions may be discovered [6,8].

In young adults, episodic spreading paresthesias more often raise suspicion for migraine aura, demyelinating disease, metabolic derangements, or transient vascular phenomena [10,11]. In this case, the patient’s sequential sensory progression, brief facial weakness, and post-event headache initially suggested a migraine variant or demyelinating process. Overall, the patient’s transient symptoms were most consistent with a migraine-spectrum phenomenon, and the subependymoma was likely an incidental finding rather than the direct cause. Early MRI was critical to exclude demyelination and identify unexpected structural lesions [5,7].

The absence of hydrocephalus or mass effect supported an indolent lesion unlikely to explain the patient’s transient symptoms. Demyelinating disease was considered unlikely given the lack of characteristic white matter lesions on MRI and a normal neurological examination between events, while a transient ischemic attack was improbable in the setting of the patient’s young age, absence of vascular risk factors, and migratory symptom evolution. While prior studies emphasize that surgical resection is curative for symptomatic lesions [2,4], in asymptomatic or minimally symptomatic patients without evidence of CSF obstruction, observation with serial imaging is a safe and effective strategy [6,8,9]. Multidisciplinary review ensured that invasive procedures such as CSF sampling or surgery were unnecessary.

## Conclusions

This case underscores the value of early MRI in young adults with episodic paresthesias, which may uncover incidental intraventricular lesions. Subependymomas can present atypically, even in the absence of obstructive symptoms, and small, non-obstructive fourth-ventricular lesions may be safely managed with conservative observation and serial imaging, demonstrating excellent short-term clinical and radiographic stability. Although longer-term follow-up is required to confirm enduring indolence. Reporting such cases enhances understanding of the variable presentations and appropriate management strategies for this rare tumor.
